# Longitudinal Associations of Structural and Functional Brain Connectivity With Dimensions of Psychopathology in Adolescence

**DOI:** 10.1016/j.bpsc.2025.09.015

**Published:** 2025-09-24

**Authors:** Lucy Vanes, Divyangana Rakesh, Tobias Banaschewski, Arun L.W. Bokde, Sylvane Desrivières, Herta Flor, Hugh Garavan, Penny Gowland, Antoine Grigis, Andreas Heinz, Herve Lemaitre, Jean-Luc Martinot, Marie-Laure Paillère Martinot, Eric Artiges, Frauke Nees, Dimitri Papadopoulos Orfanos, Luise Poustka, Michael N. Smolka, Sarah Hohmann, Nathalie Holz, Nilakshi Vaidya, Henrik Walter, Robert Whelan, Gunter Schumann, Gareth J. Barker

**Affiliations:** Department of Neuroimaging, Institute of Psychiatry, Psychology & Neuroscience, King’s College London, London, United Kingdom (LV, DR, GJB); Department of Child and Adolescent Psychiatry and Psychotherapy, Central Institute of Mental Health, Medical Faculty Mannheim, Heidelberg University, and German Center for Mental Health (DZPG), partner site Mannheim-Heidelberg-Ulm, Mannheim, Germany (TB, FN, SH, NH); Discipline of Psychiatry, School of Medicine, and Trinity College Institute of Neuroscience, Trinity College Dublin, Dublin, Ireland (ALWB); Social, Genetic and Developmental Psychiatry Centre, Institute of Psychiatry, Psychology & Neuroscience, King’s College London, London, United Kingdom (SD); Institute of Cognitive and Clinical Neuroscience, Central Institute of Mental Health, Medical Faculty Mannheim, Heidelberg University, Mannheim, Germany (HF); Department of Psychology, School of Social Sciences, University of Mannheim, Mannheim, Germany (HF, DPO); Departments of Psychiatry and Psychology, University of Vermont, Burlington, Vermont (HG); Sir Peter Mansfield Imaging Centre, School of Physics and Astronomy, University of Nottingham, University Park, Nottingham, United Kingdom (PG); NeuroSpin, Commissariat à l’Énergie Atomique et aux Energies Alternatives, Université Paris-Saclay, Gif-sur-Yvette, France (AG, HL); Department of Psychiatry and Psychotherapy, University of Tübingen, and German Center for Mental Health (DZPG), partner site Tübingen, Tübingen, Germany (AH); Institut des Maladies Neurodégénératives, Unité Mixte de Recherche 5293, Centre National de la Recherche Scientifique, Commissariat à l’Énergie Atomique et aux Energies Alternatives, Université de Bordeaux, Bordeaux, France (HL); Institut National de la Santé et de la Recherche Médicale U A10 “Trajectoires Développementales & Psychiatrie,” University Paris-Saclay, Ecole Normale Supérieure Paris-Saclay, Centre National de la Recherche Scientifique, Centre Borelli, Gif-sur-Yvette, France (J-LM, M-LPM, EA); L’Assistance Publique–Hôpitaux de Paris, Sorbonne Université, and Department of Child and Adolescent Psychiatry, Pitié-Salpêtrière Hospital, Paris, France (M-LPM); Psychiatry Department, Etablissement Public de Santé Barthélémy Durand, Etampes, France (EA); Institute of Medical Psychology and Medical Sociology, University Medical Center Schleswig Holstein, Kiel University, Kiel, Germany (FN); Department of Child and Adolescent Psychiatry, Center for Psychosocial Medicine, University Hospital Heidelberg, Heidelberg, Germany (LP); Department of Psychiatry and Psychotherapy, Technische Universität Dresden, Dresden, Germany (MNS); Centre for Population Neuroscience and Medicine Precision, Department of Psychiatry and Psychotherapy, Charité Universitätsmedizin Berlin, Berlin, Germany (NV, GS); Department of Psychiatry and Psychotherapy, Campus Charité Mitte, Charité–Universitätsmedizin Berlin, corporate member of Freie Universität Berlin, Humboldt-Universität zu Berlin, and Berlin Institute of Health, Berlin, Germany (HW); School of Psychology and Global Brain Health Institute, Trinity College Dublin, Dublin, Ireland (RW); and Centre for Population Neuroscience and Medicine Precision, Institute for Science and Technology of Braininspired Intelligence, Fudan University, Shanghai, China (GS).; The IMAGEN Consortium consists of Dr. Michael Rapp, Charité; Dr. Eric Artiges, Institut National de la Santé et de la Recherche Médicale; Sophia Schneider, University of Hamburg; Christine Bach, Central Institute of Mental Health; Prof. Dr. Tomas Paus, University of Toronto; Alexis Barbot, Commissariat à l’Energie Atomique; Prof. Dr. Gareth Barker, King’s College London; Dr. Arun Bokde, Trinity College Dublin; Dr. Nora Vetter, Technische Universität Dresden; Prof. Dr. Christian Büchel, University of Hamburg; Dr. Anna Cattrell, King’s College London; Patrick Constant, PERTIMM; Prof. Penny Gowland, University of Nottingham; Dr. Hans Crombag, University of Sussex; Katharina Czech, Charité; Dr. Jeffrey Dalley, Cambridge University; Benjamin Decideur, Commissariat à l’Energie Atomique; Dr. Tade Spranger, Deutsches Referenzzentrum für Ethik/Institut of Science and Ethics; Dr. Tamzin Ripley, University of Sussex; Dr. Nadja Heym, University of Nottingham; Prof. Herta Flor, Central Institute of Mental Health; Dr. Wolfgang Sommer, Central Institute of Mental Health; Birgit Fuchs, GABO:milliarium mbH and Co. KG; Dr. Jürgen Gallinat, Charité; Dr. Hugh Garavan, Trinity College Dublin; Prof. Dr. Rainer Spanagel, Central Institute of Mental Health; Mehri Kaviani, University of Nottingham; Dr. Bert Heinrichs, Deutsches Referenzzentrum für Ethik; Prof. Dr. Andreas Heinz, Charité; Naresh Subramaniam, Cambridge University; Dr. Tianye Jia, King’s College London; Albrecht Ihlenfeld, Physikalisch-Technische Bundesanstalt; James Ireland, Delosis; Dr. Bernd Ittermann, Physikalisch-Technische Bundesanstalt; Dr. Patricia Conrod, King’s College London; Prof. Dr. Tobias Banaschewski, Central Institute of Mental Health; Jennifer Jones, Trinity College Dublin; Dr. Arno Klaassen, Scito; Christophe Lalanne, Commissariat à l’Energie Atomique; Dr. Dirk Lanzerath, Deutsches Referenzzentrum für Ethik; Dr. Claire Lawrence, University of Nottingham; Dr. Hervé Lemaitre, Institut National de la Santé et de la Recherche Médicale; Dr. Sylvane Desrivieres, King’s College London; Catherine Mallik, King’s College London; Prof. Dr. Karl Mann, Central Institute of Mental Health; Dr. Adam Mar, Cambridge University; Lourdes Martinez-Medina, King’s College London; Prof. Dr. Jean-Luc Martinot, Institut National de la Santé et de la Recherche Médicale; Eva Mennigen, Technische Universität Dresden; Dr. Fabiana Mesquita de Carvahlo, King’s College London; Yannick Schwartz, Commissariat à l’Energie Atomique; Dr. Ruediger Bruehl, Physikalisch-Technische Bundesanstalt; Kathrin Müller, Technische Universität Dresden; Frauke Nees, Central Institute of Mental Health; Charlotte Nymberg, King’s College London; Dr. Mark Lathrop, Centre National de Genotypage; Prof. Dr. Trevor Robbins, Cambridge University; Dr. Zdenka Pausova, University of Toronto; Dr. Jani Pentilla, INSERMIERTEE; Dr. Francesca Biondo, King’s College London; Dr. JeanBaptiste Poline, Commissariat à l’Energie Atomique; Dr. Sarah Hohmann, Central Institute of Mental Health; Dr. Luise Poustka, University Medical Centre Göttingen/Medical University of Vienna; Sabina Millenet, Central Institute of Mental Health; Prof. Dr. Michael Smolka, Technische Universität Dresden; Juliane Fröhner, Technische Universität Dresden; Dr. Maren Struve, Central Institute of Mental Health; Prof. Dr. Steve Williams, King’s College London; Dr. Thomas Hübner, Technische Universität Dresden; Uli Bromberg, University of Hamburg; Semiha Aydin, Physikalisch-Technische Bundesanstalt; John Rogers, Delosis; Alexander Romanowski, Charité; Dr. Christine Schmäl, Central Institute of Mental Health; Dirk Schmidt, Technische Universität Dresden; Stephan Ripke, Technische Universität Dresden; Dr. Mercedes Arroyo, Cambridge University; Dr. Florian Schubert, Physikalisch-Technische Bundesanstalt; Dr. Yolanda Pena-Oliver, University of Sussex; Mira Fauth-Bühler, FOM Hochschule für Oekonomie and Management/Central Institute of Mental Health; Xavier Mignon, PERTIMM; Dr. Robert Whelan, Trinity College Dublin; Dr. Claudia Speiser, GABO:milliarium mbH and Co. KG; Tahmine Fadai, University of Hamburg; Prof. Dr. Dai Stephens, University of Sussex; Dr. Andreas Ströhle, Charité; Dr. Marie-Laure Paillere, Institut National de la Santé et de la Recherche Médicale; Nicole Strache, Charité; David Theobald, Cambridge University; Sarah Jurk, Technische Universität Dresden; Dr. Helene Vulser, Institut National de la Santé et de la Recherche Médicale; Ruben Miranda, Institut National de la Santé et de la Recherche Médicale; Dr. Juliana Yacubilin, University of Hamburg; Vincent Frouin, Commissariat à l’Energie Atomique; Alexander Genauck, Charité; Caroline Parchetka, Charité; Isabel Gemmeke, Charité; Johann Kruschwitz, Charité; Katharina Weiß, Charité; Dr. Henrik Walter, Charité Universitätsmedizin Berlin; Jianfeng Feng, Fudan University/Warwick University; Dimitri Papadopoulos, Institut National de la Santé et de la Recherche Médicale; Irina Filippi, Institut National de la Santé et de la Recherche Médicale; Alex Ing, King’s College London; Dr. Barbara Ruggeri, King’s College London; Bing Xu, King’s College London; Christine Macare, King’s College London; Dr. Congying Chu, King’s College London; Eanna Hanratty, King’s College London; Dr. Erin Burke Quinlan, King’s College London; Dr. Gabriel Robert, King’s College London; Prof. Dr. Gunter Schumann, King’s College London; Dr. Tao Yu, King’s College London; Veronika Ziesch, Technische Universität Dresden; Alicia Stedman, University of Nottingham.

## Abstract

**BACKGROUND:**

Adolescence is a critical period of neurodevelopment marked by ongoing maturation of structural and functional brain connectivity. Simultaneously, this period is associated with an increase in mental health problems, spanning from subclinical symptoms to diagnosable disorders.

**METHODS:**

This study investigated longitudinal associations between psychopathology dimensions and voxelwise brain measures related to connectivity across 3 time points (ages 14, 19, and 23) in more than 1500 participants using the IMAGEN dataset. White matter (WM) microstructure was indexed using diffusion metrics quantified along the WM skeleton (*N* = 1736), while functional connectivity was captured as voxelwise degree centrality (DC) derived from resting-state functional imaging (*N* = 1510).

**RESULTS:**

Development of WM microstructure was selectively linked to externalizing (but not internalizing) symptoms. Here, higher externalizing symptoms were associated with widespread reductions in fractional anisotropy (FA) across the WM skeleton as well as accelerated decreases in FA in the corticospinal tract over time. In contrast, functional DC was developmentally associated with general, rather than specific, psychopathology in frontal and temporal regions. An increase in total difficulties over time was associated with developmental decrease in DC in bilateral superior frontal gyri. In addition, a positive association between total difficulties and DC in left inferior temporal gyrus was observed in younger, but not older, adolescents or young adults.

**CONCLUSIONS:**

These findings highlight the dynamic interplay between brain connectivity development and psychopathology in adolescence, with potential implications for identifying neural markers of risk and resilience during sensitive windows of development.

Connectivity underpins the functional capacities of the brain, supported by both the structural integrity of white matter (WM) tracts and the fine-tuning of functional networks (1). While structural and functional brain connectivity undergo continued development throughout the lifespan (2,3), adolescence represents a particularly sensitive period of neural reorganization (4) driven by increasing environmental demands coupled with biological processes of maturation. As a result, the manner in which neurodevelopment unfolds during this stage can have profound effects on individual cognitive, emotional, and behavioral trajectories (5).

Adolescence is also marked by increased mental health problems, ranging from diagnosable disorders (6) to subclinical difficulties that signal vulnerability (7,8). Internalizing (broadly emotional) and externalizing (broadly behavioral) problems typically rise throughout adolescence, albeit with marked heterogeneity (9–11). This underscores the need to consider their dynamic nature in studies of neurobiological etiology. Furthermore, given the potential reciprocal influences between internalizing and externalizing dimensions (12,13), it is crucial to investigate the effects of one in the context of the other.

Accumulating evidence supports an association between alterations in brain connectivity and adolescent psychopathology (14–16). An emerging picture suggests that delayed or disrupted maturation of large-scale networks (including default mode, executive, and sensorimotor) may contribute (17), whereby disrupted structural connectivity might constrain the development of stable and flexibly interconnected functional networks (18). This highlights the importance of considering both structural and functional aspects of brain development when studying the neurodevelopmental correlates of psychopathology. Diffusion imaging sheds light on microstructural WM parameters that support interregional communication, while resting state functional imaging captures evolving patterns of functional connectivity. Studying both in the same individuals, especially longitudinally, enables a more complete understanding of how mechanisms supporting brain connectivity evolve in relation to emerging mental health symptoms.

Notably, most prior neuroimaging research has relied on cross-sectional designs, potentially overlooking dynamic associations evolving over time. Where longitudinal associations have been investigated [e.g. (19,20)], work frequently focuses on predefined regions, tracts, or networks. This approach poses challenges for capturing longitudinal associations, as structural and functional network configurations themselves may change over time (21,22). This highlights a need for fine-grained (i.e., voxelwise) analyses capturing coupled evolution of brain connectivity and mental health symptoms, embracing the interindividual variability inherent in both neural and behavioral development.

In this study, we investigated longitudinal associations of psychopathology dimensions in adolescence and early adulthood with structural and functional brain connectivity. We leveraged data from the large longitudinal IMAGEN dataset (23) to assess WM microstructure using diffusion imaging and functional connectivity using resting-state functional magnetic resonance imaging (MRI) in 1736 young people ages 14 to 23 years. IMAGEN work has previously illuminated adolescent processes such as reinforcement learning (24), emotion regulation (25), substance use (26), and specific clinical traits (27), but no study has jointly examined the longitudinal development of WM and functional connectivity at the voxelwise level or linked these trajectories to broad psychopathology dimensions.

Alongside voxelwise diffusivity measures, we derived voxelwise degree centrality (DC), a whole-brain index of functional coupling of each voxel with the rest of the brain that is well suited to longitudinal analyses in which network organization may shift over time. Combined, these metrics provide holistic insight into connectivity development, unconstrained by predefined network hypotheses. After examining longitudinal associations between overall psychopathology and brain measures, we investigated the respective contribution of internalizing and externalizing dimensions—each in the context of the other—to better understand which aspects drive observed associations. Our modeling statistically disentangled within-subject developmental effects from between-subject age differences, reducing the risk of conflating individual developmental trajectories with stable differences between participants.

## METHODS AND MATERIALS

### Participants and Procedure

Participants were drawn from the European imaging genetics project IMAGEN, a multicenter longitudinal study that recruited approximately 2000 typically developing adolescents across 4 assessment waves, of which 3 (baseline: age 14; follow-up 2: age 19; follow-up 3: age 23) included MRI. The current study focused on these 3 assessments. For a detailed description of recruitment and assessment, see Schumann *et al.* (23).

After quality control, the present study included data from 1736 adolescents with diffusion data from at least 1 time point (3278 scans) and 1510 adolescents with resting-state data from at least 1 time point (2434 scans) who also had complete psychopathology data at the time of each scan (see Supplement and Table 1 for more details on scan availability by time point and modality). The study was previously approved by the local ethics committees, and participants provided written informed consent or assent (for participants <18 years of age, their legal guardians provided written informed consent). Exclusion criteria included current bipolar disorder; schizophrenia; and major neurodevelopmental conditions such as autism, preterm birth, head trauma, or history of neurological or medical disorders.

### Psychopathology

Psychopathology was assessed by self-report on the Strengths and Difficulties Questionnaire (SDQ), a 25-item scale assessing behavioral and emotional difficulties (28), administered at each study time point. Total difficulties scores, internalizing difficulties scores, and externalizing difficulties scores were derived for all participants at each time point (ages 14, 19, and 23); see Supplement for details.

### Imaging Acquisition and Preprocessing

MRI data were acquired on 3T scanners across IMAGEN sites (Siemens; Philips; General Electric) at ages 14, 19, and 23. Detailed acquisition parameters for structural T1-weighted, diffusion-weighted, and resting-state images are presented in the Supplement. Effects of site on mean fractional anisotropy (FA) and mean DC for each assessment time point are summarized in Table S7. In all analyses, site was included as a covariate of no interest to adjust for potential intersite differences. Preprocessing steps for the diffusion and resting-state data followed standard procedures of the IMAGEN Consortium; details are in the Supplement.

### Calculation of Functional Voxelwise DC

To derive voxelwise relative DC maps for each participant at each time point, the time series from each voxel within a gray matter mask was extracted and a correlation matrix constructed from pairwise Pearson correlations between each pair of voxels. Correlations below 0.2 were excluded to avoid including spurious connections. Weighted DC was calculated for each voxel as the sum of correlations between this voxel and all other voxels within the mask, reflecting, in absolute terms, the centrality of each voxel within the gray matter mask. To enhance comparability across participants and time points, relative DC maps were generated by standardizing DC values to within-subject and within–time point *z* scores. Maps thus reflect the centrality of each voxel relative to all other gray matter voxels at the time of the scan, while reducing scanner-related global differences. Longitudinal analyses of DC therefore reflect changes in the relative centrality of each voxel over time. DC was chosen as our primary resting-state metric because it provides a whole-brain, voxelwise measure of global functional connectivity without constraining analyses to predefined network boundaries. This makes it particularly well suited for longitudinal analyses, in which functional networks may reorganize over time.

### Statistical Analysis

Skeletonized diffusion metric maps (FA, trace [TR], radial diffusivity [RD], axial diffusivity [AD]) and functional connectivity maps (DC) underwent separate but identical voxelwise longitudinal analyses using nonparametric mixed-effects marginal modeling with the FSL Sandwich Estimator (SwE) tool (29) (see Supplement for more details), testing the longitudinal development of each brain metric and its association with psychopathology. Statistical inferences for effects of interest were achieved using a nonparametric wild bootstrap procedure with 999 iterations (30). Threshold-free cluster enhancement (31) was used for inference on diffusion maps to detect focal effects along the WM skeleton while controlling for familywise error rate (FWER) at *p* < .05 (2-tailed). Cluster-extent correction was used for inference on functional maps to identify spatially contiguous regions of significant effect, reflecting the typically broader, network-level changes observed in functional connectivity analyses, while also controlling for FWER. For these analyses, a cluster-forming parametric uncorrected *p*-value threshold of .001 was applied, with clusters retained at FWER-corrected *p* < .05.

For each modality, a first model including SDQ total (SDQ-TOT) scores was run, followed by a second model including both SDQ internalizing (SDQ-INT) and SDQ externalizing (SDQ-EXT) scores to clarify the relative contributions of these 2 symptom domains to any observed associations (described in more detail below). These 2 models were considered part of a planned, hierarchical analysis within each modality, with the second model (SDQ-INT and SDQ-EXT) serving as a decomposition of the first (SDQ-TOT). As they were not treated as independent tests, no additional correction was applied across them. Similarly, we did not correct across modalities, as these analyses were considered separate families of tests.

#### Longitudinal and Cross-Sectional Variable Decomposition.

The time-varying variables age at scan and SDQ-TOT, SDQ-INT, and SDQ-EXT scores were each decomposed into their cross-sectional and longitudinal components to distinguish between-subject and within-subject effects (32). This allows for a true separation of developmental effects occurring between individuals (cross-sectional) from those occurring within individuals over time (longitudinal), reducing the risk of confounding between the 2 sources of variation.

Cross-sectional age was each participant’s mean age across( time points centered) with respect to the whole sample $\left(\bar{{\text{Age}}_{\text{subject}}}-\bar{{\text{Age}}_{\text{sample}}}\right)$. A main effect of cross-sectional age reflects between-subject brain differences between (on average) younger and older participants. A histogram of mean subject age in Figure S1 shows that there was indeed meaningful variation in the average subject age, likely due to the fact that not all participants completed all scan time points.

Longitudinal age was the within-subject centered age at each time point $\left({\text{Age}}_{\text{subject},\text{time point}}-\bar{{\text{Age}}_{\text{subject}}}\right)$, henceforth referred to as time, as it represents the within-subject change over time while taking into account the temporal distance between scans. A main effect of time reflects longitudinal brain changes that occur within a subject.

Similarly, cross-sectional SDQ scores $\left(\bar{{\text{SDQ}}_{\text{subject}}}-\bar{{\text{SDQ}}_{\text{sample}}}\right)$ represent each subject’s average SDQ score across time points, while longitudinal SDQ $\left({\text{SDQ}}_{\text{subject},\text{time point}}-\bar{{\text{SDQ}}_{\text{subject}}}\right)$ represents the within-subject change in SDQ score over time. A main effect of cross-sectional SDQ reflects average brain difference between individuals experiencing different levels of psychopathology, while a main effect of longitudinal SDQ reflects within-subject coupled changes over time in psychopathology and brain metrics. SDQ-TOT, SDQ-INT, and SDQ-EXT scores were each decomposed in this manner.

#### SDQ-TOT Analysis.

The first model run for each brain modality (as a voxelwise time-varying outcome) included the following regressors: scan site, sex, cross-sectional age, time, cross-sectional SDQ-TOT, longitudinal SDQ-TOT, cross-sectional age × time (testing nonlinear developmental effects), cross-sectional age × cross-sectional SDQ-TOT (testing the interaction between average age and average SDQ-TOT score), and time × cross-sectional SDQ-TOT (testing whether longitudinal developmental effects differ as a function of average SDQ total score). For DC analyses, each participant’s mean FD was included as a further covariate.

#### SDQ-INT and SDQ-EXT Analysis.

A second model was run for each brain modality including the following regressors: scan site, sex, cross-sectional age, time, cross-sectional SDQ-INT, cross-sectional SDQ-EXT, longitudinal SDQ-INT, longitudinal SDQ-EXT, cross-sectional age × time, cross-sectional age × cross-sectional SDQ-INT, cross-sectional age × cross-sectional SDQ-EXT, time × cross-sectional SDQ-INT, and time × cross-sectional SDQ-EXT. Mean FD was included as an additional covariate in DC analyses only.

#### Sensitivity Analyses.

Additional sensitivity analyses were run to test the robustness of the observed effects. All reported effects remained significant across all sensitivity analyses; details are in the Supplement.

## RESULTS

Sample characteristics are in Table 1.

### Structural Connectivity Findings (FA, TR, RD, AD)

#### Developmental Effects.

Developmental effects were extracted from the main effects of cross-sectional age and time, as well as their interaction, within the first (SDQ-TOT) model run for each modality. Widespread developmental increases in FA were evident across the WM skeleton both cross-sectionally (positive effect of cross-sectional age), reflecting higher FA in older compared with younger participants (Figure 1A), and longitudinally (positive effect of time), reflecting a within-subject increase in FA over time (Figure 1B). In contrast, there were focal decreases in FA primarily in bilateral corticospinal tracts (CSTs), which were again reflected both cross-sectionally and longitudinally. Full cluster details are listed in Table S2. These developmental effects were largely mirrored by cross-sectional and longitudinal effects on TR, RD, and AD (Figure S2). There was no significant cross-sectional age × time interaction on any of the diffusion metrics, indicating largely linear developmental effects such that change over time did not differ as a function of age within the sample.

#### Psychopathology Effects.

FA analyses revealed a significant negative main effect of cross-sectional SDQ-TOT across large parts of the WM skeleton including corpus callosum, bilateral anterior thalamic radiation, bilateral CSTs, and bilateral inferior fronto-occipital fasciculi (Figure 2A; Table S3), indicating that a higher average SDQ-TOT score across time points was associated with widespread lower average FA across time points (Table S3). This effect was reflected in more focal effects for AD and, to a lesser extent, TR (Figure 2A). There was no effect of cross-sectional SDQ-TOT on RD.

Models including both SDQ-INT and SDQ-EXT scores revealed that the observed SDQ-TOT effects were mirrored in a significant negative main effect of cross-sectional SDQ-EXT on widespread FA, as well as TR and AD (Figure 2B; Table S4). In contrast, there was no main effect of cross-sectional SDQ-INT on any of the diffusion metrics. In addition, there was a significant negative time × cross-sectional SDQ-EXT interaction on FA in left CST, indicating that participants with higher externalizing symptoms demonstrated greater decreases in FA over time in this tract (Figure 3; Table S5). This effect was not reflected in TR, RD, or AD.

### Degree Centrality

#### Developmental Effects.

There were no significant main effects or interaction of cross-sectional age or time on DC, indicating that there was no systematic (cross-sectional or longitudinal) developmental effect in terms of relative voxelwise connectivity.

#### Psychopathology Effects.

There was a significant negative effect of longitudinal SDQ-TOT, indicating that an increase in SDQ-TOT score over time was associated with a reduction of DC primarily in bilateral posterior superior frontal gyri (pSFG), extending to precentral gyri, over time (Figure 4; Table S6). Finally, there was a significant cross-sectional age × cross-sectional SDQ-TOT interaction in the left inferior temporal gyrus (ITG), indicating that, in younger participants, higher average SDQ-TOT scores were associated with increased average ITG connectivity. This association was not present in older participants (Figure 5; Table S7).

## DISCUSSION

This study examined longitudinal associations between structural and functional brain development and psychopathology in adolescence and young adulthood using a large, population-based sample. We observed that WM microstructural development was selectively associated with externalizing symptoms, with higher symptom levels linked to both widespread lower FA and steeper declines of FA over time in the left CST. In contrast, functional brain connectivity, indexed by voxelwise DC, showed effects linked to overall psychopathology, without strong differentiation between internalizing and externalizing symptoms. Longitudinal increases in total difficulties were associated with lower superior frontal connectivity, and younger adolescents showed a selective association between total difficulties and inferior temporal connectivity. These results highlight distinct but complementary patterns across structural and functional domains, suggesting that specific features of brain development are differentially sensitive to distinct aspects of emerging psychopathology during adolescence.

Structural neuroimaging research on developmental mental health has previously highlighted broad WM involvement. Lower global WM microstructure has been associated with higher general psychopathology in children (33), while widespread alterations across multiple WM tracts have been linked to psychopathology in adolescents (16,34,35). Our findings in the IMAGEN cohort reflect this, showing widespread cross-sectional negative associations between FA and total difficulties. Corresponding patterns in TR and AD—though not RA—point to variation in axonal properties rather than differences in myelination as driving this effect (36,37). Notably, our follow-up analyses suggest that this brain-behavior relationship is primarily accounted for by externalizing rather than internalizing symptoms, aligning with previous work linking WM microstructure to disruptive behaviors in youth (38,39).

In addition to these widespread effects, we identified a more focal longitudinal association in the left CST, in which higher externalizing symptoms were linked to greater reductions in FA over time. This tract, traditionally associated with motor control (40), has increasingly been implicated in behavioral regulation (41) as well as higher-order processes (42). Similar longitudinal patterns have been reported in adolescents with attention-deficit/hyperactivity disorder (ADHD), in which atypical CST development was linked to persistent behavioral problems (43). Conversely, clinical improvement of hyperactivity-impulsivity symptoms in ADHD was associated with increased CST fiber density in young adulthood (44). Our work extends this by showing that even in a community sample, variation in CST maturation tracks externalizing symptoms, potentially reflecting delayed or disrupted integration of motor-executive pathways involved in impulse control.

Focusing on brain function, we found a longitudinal coupling between superior frontal connectivity and general psychopathology, in which reduced centrality of bilateral pSFG over time was associated with a symptom exacerbation from ages 14 to 22. DC reflects the extent to which a brain region is functionally connected to the rest of the brain, serving as a marker of its integrative role in large-scale brain communication. The SFG is a key hub within the frontoparietal and default mode networks, and its connectivity is thought to support cognitive control (45), emotion regulation (46), and broader executive functioning (47). pSFG more specifically has been implicated in sociocognitive processes such as mentalizing (48,49), and diminished default mode connectivity within this region has been linked to greater social dysfunction in individuals with major depressive disorder (50). Given the ongoing maturation of prefrontal circuits during adolescence, declining pSFG centrality may reflect an altered neurodevelopmental trajectory that compromises regulatory or socioemotional capacity, contributing to increased nonspecific psychiatric vulnerability. Previous research supports this, linking superior frontal dysconnectivity to affective symptoms (51,52), cognitive impairments (53), and psychotic-like experiences (54). These findings suggest that developmental changes in pSFG connectivity might serve as a sensitive marker for emerging mental health difficulties.

We further observed an age-specific effect linking general psychopathology to inferior temporal connectivity: Younger, but not older, adolescents showed a positive relationship between total difficulties and left ITG centrality. Left ITG has been linked to cognitive functions such as language (55), visual perception (56), and memory (57) and, more recently, emotion regulation (58,59)—all of which can be disrupted in youth with elevated psychopathology (60). Neuroimaging studies report altered ITG function and structure in relation to internalizing (61–63) or externalizing (64,65) symptoms, or both (66), though inconsistencies remain with respect to the timing and direction of effects. Our findings may help explain this variability, suggesting a compensatory mechanism at younger ages, whereby increased ITG connectivity supports emotional or sensory processing in the face of emerging difficulties. This interpretation aligns with evidence that enhanced left ITG connectivity may play a compensatory role that buffers against the impact of childhood trauma on risk for developing depression later in life (67). The reduction of the association between ITG connectivity and psychopathology in older adolescents could reflect a diminished reliance on, or capacity for, this compensatory recruitment as cognitive and neural systems mature. However, it is worth noting that this interaction was not reflected longitudinally (i.e., the ITG-psychopathology association did not decrease within subjects over time), suggesting the effect may reflect differences between age groups rather than true developmental change. Future work should test whether the observed effect is mediated by social or environmental factors that vary across age groups or reflects a time-limited neural strategy that supports regulation in the face of early symptoms.

Of note, sensitivity analyses showed that all effects remained significant after controlling for socioeconomic status and including interactions with sex. While this strengthens robustness, future work should directly test sex-specific and socioenvironmental influences given their importance in adolescent psychopathology. Our findings suggest that structural and functional brain changes show distinct but complementary associations with adolescent mental health, with WM more closely tied to externalizing symptoms and functional connectivity more closely tied to general psychopathology. This aligns with emerging models that emphasize both domain-specific and transdiagnostic neurobiological processes (68–70). However, it is also important to consider that structural and functional connectivity may show bidirectional influences over time, whereby WM architecture constrains functional brain network maturation (71), and functional activity shapes structural refinement through experience-dependent plasticity (72). Increased structure-function coupling has been linked to increased negative affectivity (73), suggesting that greater structural constraints on function may result in adverse psychological outcomes. At the same time, while bidirectional influences may exist at the biological level, our findings indicate that associations with psychopathology can be modality specific. Structure-function coupling is dynamic across development (74), with the developmental rate of this coupling itself associated with behavioral outcomes in adolescence (75). Moreover, structure and function may influence each other at different points in time, such that structural and functional pathways exert their effects on psychopathology at different stages of development, potentially explaining modality-specific associations observed in our study. Further work is needed to test how structure-function coupling contributes to emerging psychopathology and whether distinct structural and functional pathways converge or operate independently across symptom dimensions.

A separate analysis of structural and functional brain development in relation to psychopathology is also warranted given our observation of differing developmental patterns. While diffusion measures were highly sensitive to broad developmental effects, DC did not show comparable generalized age effects. This divergence may reflect differences in the developmental trajectories of structural and functional connectivity, with WM microstructure following more consistent age-related patterns, whereas functional DC may be more variable and context dependent (76). This contrasts with previous work showing normative age-related changes in functional connectivity (77,78), which were not reflected in our data. This discrepancy is likely due to differences in the specific measures of functional connectivity used across studies. Voxelwise relative DC has not, to our knowledge, been examined longitudinally in the age range studied here; the absence of normative developmental effects in our sample is therefore informative. It is possible that this developmental window represents a plateau phase in functional centrality, consistent with reports of increases in (parcellation-based) DC in younger cohorts (79) and decreases in DC in older cohorts (80). While DC did not show normative age effects in our study, it was sensitive to symptom-related changes as well as age-specific symptom associations, supporting its value for capturing clinically relevant variability. Together, these findings highlight the need to consider modality-specific developmental dynamics when interpreting brain-behavior associations. Here, gray matter morphometry may provide further complementary insights beyond brain connectivity, shedding light on the extent to which cortical and subcortical structural changes parallel or diverge from connectivity-based associations with psychopathology.

This study benefits from a large, longitudinal dataset and voxelwise analytic approaches that allow for fine-grained detection of developmental change. A key strength is the decomposition of within- and between-subject effects, helping to minimize conflation of cohort differences with true developmental change, a common limitation in adolescent research. However, there are also caveats to consider. The SDQ, while practical and widely validated, provides only a brief dimensional snapshot of psychopathology and may not fully capture clinical complexity (81). Our findings may have been impacted by the specific sample makeup of the IMAGEN cohort. For example, the relatively low average severity of psychopathology in this community-based sample may have limited sensitivity to detect associations that might be more pronounced in higher-risk clinical populations. DTI metrics such as FA are also inherently nonspecific and cannot unambiguously distinguish between biological processes (82). DC reflects the relative importance of a node within the network based on number of connections, but does not capture absolute connectivity strength and or account for known large-scale network organization, limiting interpretability in terms of specific functional systems. Despite these limitations, combining DTI and DC measures provides complementary insights into brain development, capturing microstructural and functional connectivity patterns linked to emerging mental health symptoms. Applied within a large, longitudinal cohort with voxelwise resolution and within-subject modeling, this approach offers a robust, data-driven characterization of neurodevelopmental risk.

Adolescence is a sensitive window for brain development during which connectivity patterns evolve alongside increasing psychopathology. Our findings suggest that externalizing symptoms are particularly linked to WM microstructure, while general psychopathology is associated with dynamic changes in functional connectivity. These results highlight the importance of distinguishing between domain-specific and transdiagnostic neurodevelopmental pathways, advancing our understanding of the neural mechanisms underlying diverse psychiatric outcomes.

## Supplementary Material

1

## Figures and Tables

**Figure 1.**
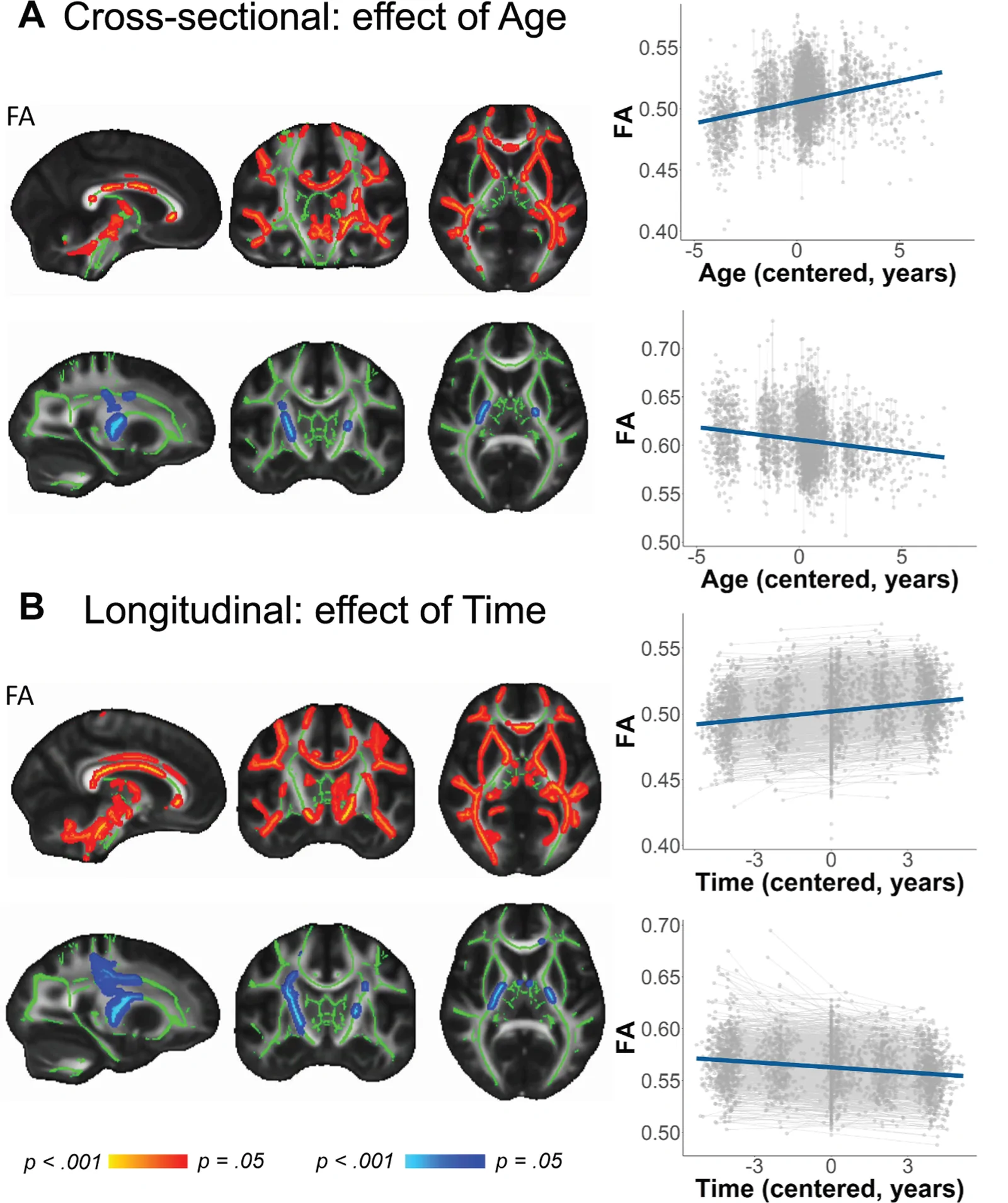
Positive (red) and negative (blue) developmental effects on fractional anisotropy (FA), captured cross-sectionally **(A)** and longitudinally **(B).** Scatterplots show mean FA across all voxels within the significant clusters displayed in the corresponding brain maps.

**Figure 2.**
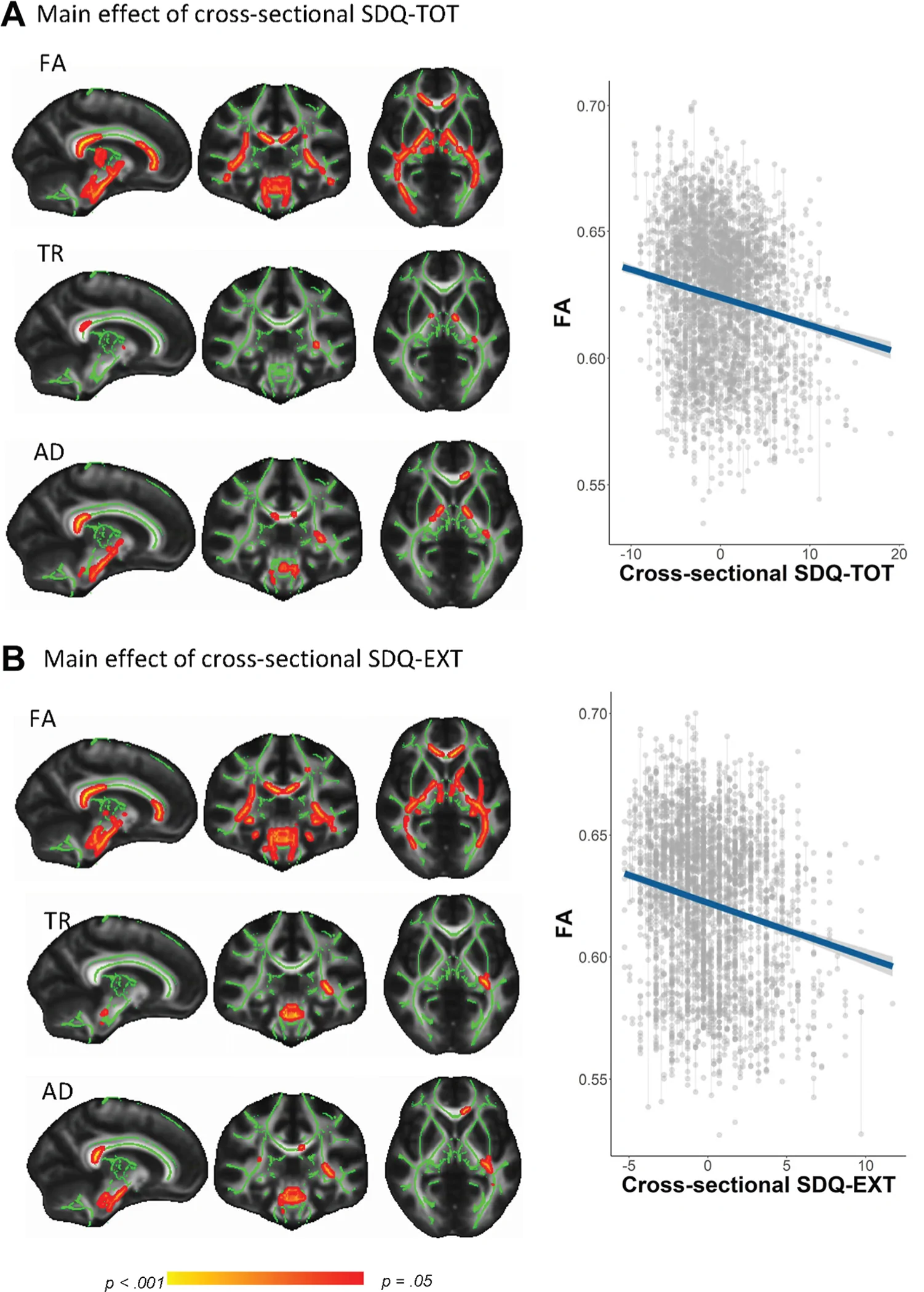
Effect of average Strengths and Difficulties Questionnaire total (SDQ-TOT) score **(A)** and SDQ externalizing (SDQ-EXT) score **(B)** on fractional anisotropy (FA), trace (TR), and axial diffusivity (AD). Scatterplots show mean FA across all voxels within the significant clusters displayed in the corresponding brain maps.

**Figure 3.**
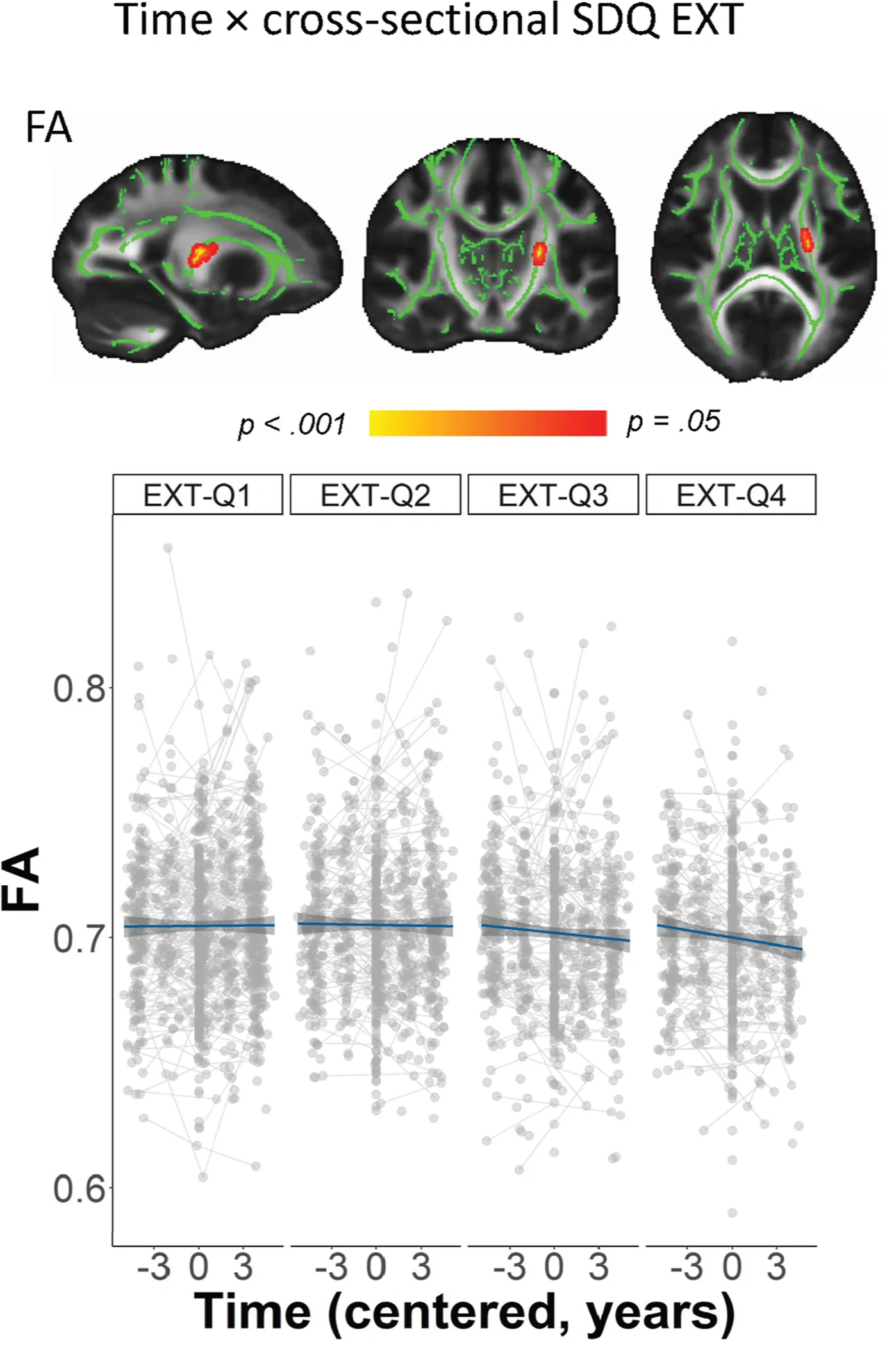
Change in fractional anisotropy (FA) over time as a function of average Strengths and Difficulties Questionnaire externalizing (SDQ-EXT) score. Scatterplot depicts mean FA across all voxels within the significant clusters displayed in the brain map. For visualization only, SDQ-EXT score is divided into quartiles (EXT-Q1 to EXT-Q4). Time reflects within-subject centered age, representing the deviation from each participant’s mean age across time points. FA in corticospinal tract decreases most rapidly in participants with highest average SDQ-EXT scores.

**Figure 4.**
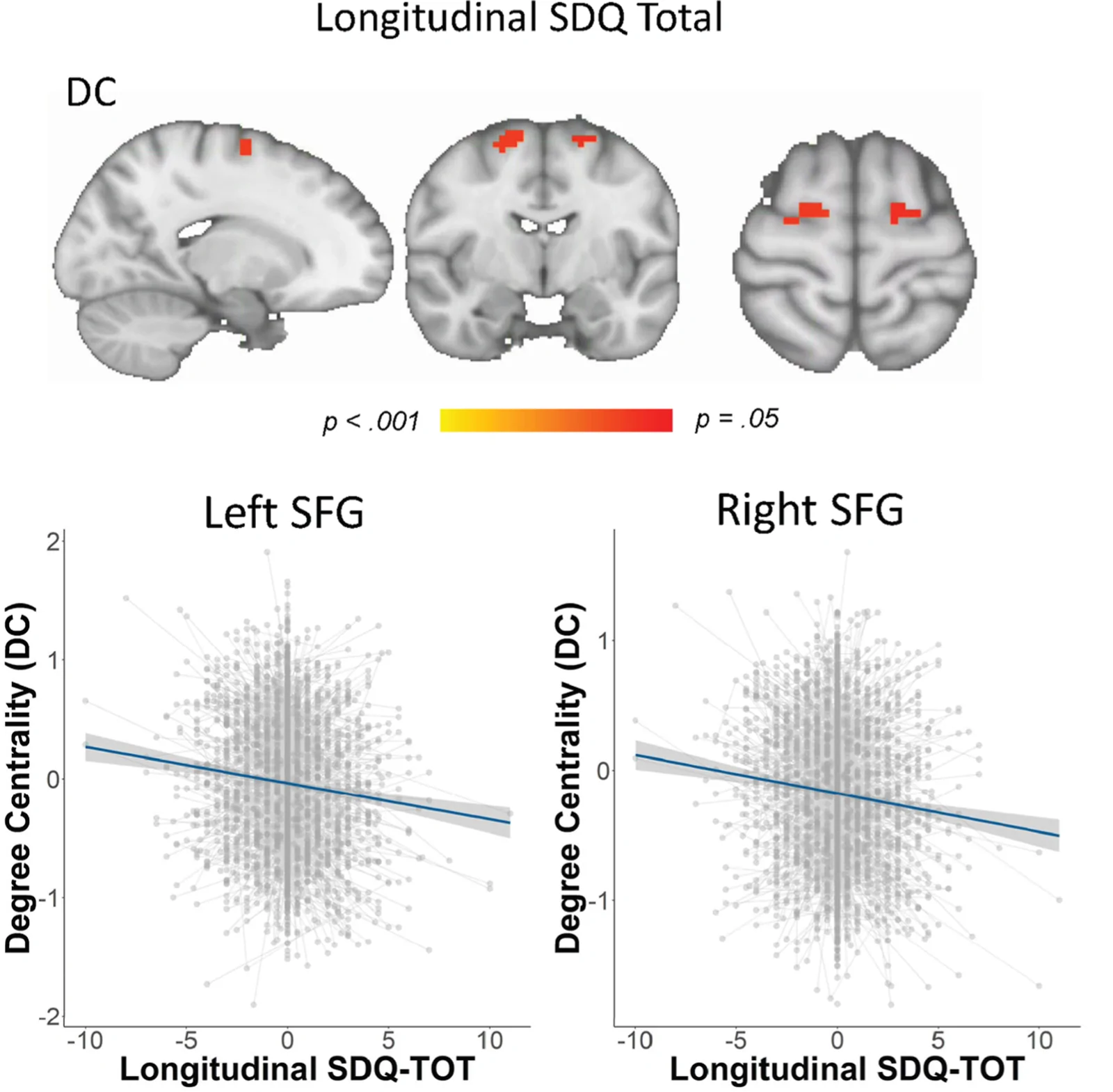
Effect of longitudinal Strengths and Difficulties Questionnaire total (SDQ-TOT) score on degree centrality (DC) in bilateral superior frontal gyri (SFG). Scatterplots depict mean DC within each significant cluster. Within-subject increase in total score is associated with within-subject DC decrease in SFG.

**Figure 5.**
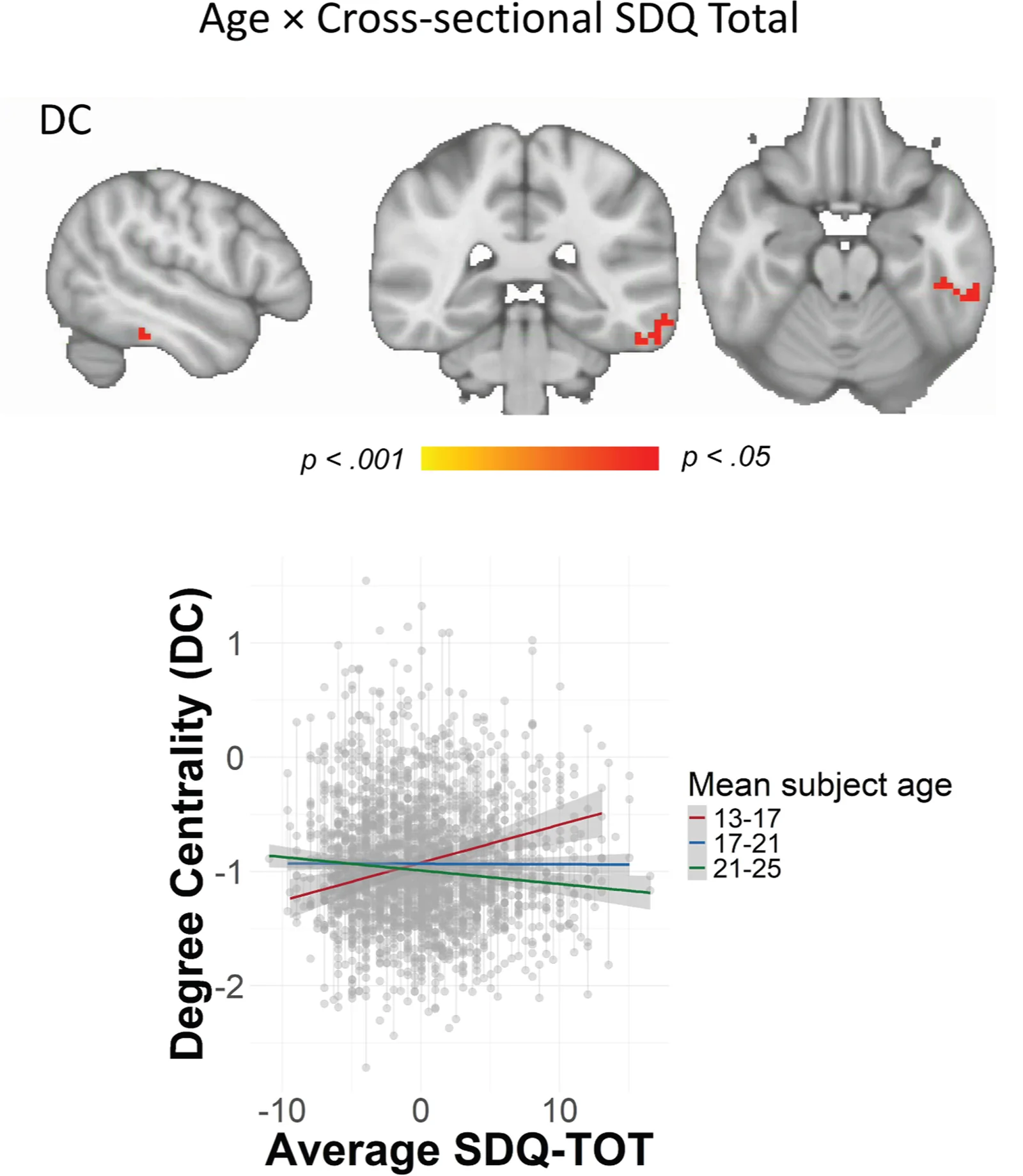
Age by Strengths and Difficulties Questionnaire total (SDQ-TOT) score interaction on degree centrality (DC) in left inferior temporal gyrus (ITG). Scatterplot depicts mean DC within the significant cluster. For visualization only, participants are grouped into age bands (13–17, 17–21, 21–25) based on cross-sectional age, reflecting each participant’s mean age across available time points. Age bands do not correspond directly to assessment waves. Higher total difficulties are associated with ITG hyperconnectivity in younger participants.

**Table 1.** Sample Characteristics by Assessment Time Point

Variable	Baseline	FU2	FU3
No. of Scans			
DTI	1411	943	924
fMRI	331	988	1115
Age, Years			
DTI sample			
Mean (SD)	14.44 (0.40)	19.01 (0.71)	22.54 (0.65)
Median	14.42	18.82	22.40
Range	13.23–15.72	17.82–21.53	20.73–25.11
fMRI sample			
Mean (SD)	14.48 (0.40)	19.01 (0.72)	22.55 (0.65)
Median	14.47	18.82	22.42
Range	13.52–15.48	17.82–21.53	20.89–25.13
Sex, Female, %			
DTI sample	52.7%	53.4%	53.7%
fMRI sample	46.5%	53.0%	53.3%
SES, Mean (SD)			
DTI sample	0.68 (1.07)		
fMRI sample	0.60 (0.94)		
SDQ Score, Mean (SD)			
DTI sample			
SDQ-TOT	17.98 (4.58)	18.24 (4.94)	17.73 (4.73)
SDQ-INT	4.37 (2.89)	4.67 (3.14)	4.45 (3.09)
SDQ-EXT	5.89 (3.09)	5.08 (2.81)	4.54 (2.62)
fMRI sample			
SDQ-TOT	18.16 (4.73)	18.35 (4.97)	17.54 (4.68)
SDQ-INT	4.57 (3.05)	4.78 (3.19)	4.40 (3.05)
SDQ-EXT	5.99 (3.09)	5.07 (2.80)	4.56 (2.61)

DTI, diffusion tensor imaging; fMRI, functional magnetic resonance imaging; FU2, second follow-up of IMAGEN study; FU3, third follow-up of IMAGEN study; SDQ, Strengths and Difficulties Questionnaire; SDQ-EXT, SDQ externalizing; SDQ-INT, SDQ internalizing; SDQ-TOT, SDQ total; SES, socioeconomic status.
