# Erratum to: Longitudinal Associations of Structural and Functional Brain Connectivity With Dimensions of Psychopathology in Adolescence

**DOI:** 10.1016/j.bpsc.2026.07.011

**Published:** 2026-08

Erratum to: “Longitudinal Associations of Structural and Functional Brain Connectivity With Dimensions of Psychopathology in Adolescence”, by Vanes *et al.* (*Biol Psychiatry Cogn Neurosci Neuroimaging* 2026; 11: 963–975); https://doi.org/10.1016/j.bpsc.2025.09.015.

The surname for coauthor Arun L.W. Bokde has been corrected.

