# Supplementary material for: Longitudinal Associations of Structural and Functional Brain Connectivity With Dimensions of Psychopathology in Adolescence
Source: Biol Psychiatry Cogn Neurosci Neuroimaging. Author manuscript; Available in PMC 2026 Oct 8. (PMC13646494; doi:10.1016/j.bpsc.2025.09.015)
Supplement: Supplementary file 1 [file NIHMS2202782-supplement-1.pdf]

SUPPLEMENTARY INFORMATION

Longitudinal Associations of Structural and Functional Brain Connectivity With Dimensions of Psychopathology in Adolescence

Vanes *et al.*

Supplemental Information

IMAGEN sample

Participants were drawn from the European Imaging Genetics (IMAGEN) project, a multi-centre longitudinal study that recruited ~2000 typically developing adolescents across four assessment waves, of which three (baseline: age 14; follow-up 2: age 19; follow-up 3: age 23) included MRI sessions at age 14, 18, and 22. The current study focused on these three assessments. For a detailed description of recruitment and assessment procedures, refer to Schumann et al (2010). Briefly, IMAGEN recruited a population-based sample designed to maximise variability in behavioural and emotional functioning, while also limiting population stratification effects for genetic analyses. Participants with major neurodevelopmental conditions such as autism or serious mental health disorders requiring treatment (schizophrenia or bipolar disorder) were excluded at baseline to minimise potential confounds associated with severe clinical conditions that could disproportionately influence brain-behaviour associations. The cohort therefore reflects a broad spectrum of subclinical psychopathology and socio-demographic backgrounds, with diversity in socioeconomic status, academic achievement, and behavioural/emotional functioning.

Imaging acquisition

MRI data were acquired on 3T scanners across IMAGEN sites (Siemens; Philips; General Electric) at age 14, 18, and 22.

High resolution structural T1-weighted images were acquired with a magnetization prepared rapid acquisition echo (MPRAGE) sequence based on the ADNI protocol.

Diffusion-weighted images were acquired using an echo planar imaging sequence with identical protocols across sites with the following parameters: 4 b-value = 0 s/mm2 and 32 diffusion encoding directions with b-value = 1300 s/mm2; TR/TE = 1500 ms/104 ms, voxel size = 2.4 mm isotropic, 128x128 matrix, field of view 307x307 mm; 60 oblique axial slices parallel to the anterior commissure/posterior commissure line.

Resting-state images were acquired using blood oxygen level-dependent contrast with the following parameters: TR/TE = 2200/30 ms; flip angle = 75°; 40 parallel slices; fat suppression; voxel size = 3.4 × 3.4 × 2.4 mm. A total of 164 (N = 1460 scans) or 187 (N = 974 scans) volumes were collected (differing by site), with the maximum number of volumes used for analysis. Participants were asked to close their eyes throughout the scan.

Scan availability

The present study included data from 1736 adolescents with diffusion data from at least one timepoint (3278 scans total). This included 655 participants with one scan, 620 participants with scans from two timepoints, and 461 participants with all three scans. Resting-state data was available from 1510 adolescents from at least one timepoint (2434 scans total), including 695 participants with one scan, 706 with scans at two timepoints, and 109 participants with scans from all three timepoints.

Diffusion imaging data processing

Diffusion images were pre-processed using MRtrix3, including denoising, removal of Gibbs ringing artifacts, motion and distortion correction, and bias field correction. They were then longitudinally pre-processed using a tensor-based registration with DTI-TK (http://dti-tk.sourceforge.net/pmwiki/pmwiki.php) (2). Within-subject templates were first created based on each subject's images across timepoints, followed by creation of a group-wise atlas based on these templates (3). Each participant's diffusion data were then normalised to the MNI standard space using the IIT human brain tensor template (4). Registered tensor images were used to compute voxelwise diffusion metrics, including fractional anisotropy (FA), trace (TR), axial diffusivity (AD), and radial diffusivity (RD), using DTI-TK. Registered images then underwent a tract-based spatial statistics (TBSS) procedure (5) to enable voxel-wise statistical inference of diffusion tensor metrics. A mean FA image was derived and thinned to create a mean FA skeleton, thresholded at FA > 0.2. Each participant's FA, TR, AD and RD data were then projected onto the common skeleton. FA served as the primary diffusion metric of interest, with TR, AD, and RD serving to aid interpretation of any FA effects by providing complementary information on overall diffusivity, as well as diffusion parallel and perpendicular to the principal fibre orientation. Visual quality control procedures were conducted at each registration step to discard images with defective spatial normalisation.

Resting-state data pre-processing

Preprocessing of resting-state data was performed with routines from FMRIB's Software Library (FSL v5.0.9) and Advanced Normalization Tools (ANTs v1.9.2). Motion correction was carried out, applying a rigid body registration of each volume to the middle volume (FSL MCFLIRT). Non-brain tissue was removed (FSL BET). Spatial smoothing was applied using a 4mm FWHM Gaussian kernel. Independent component analysis (FSL MELODIC) was run for each data set. Artifact components were identified using an automatic classification algorithm and subsequently regressed from the data (ICA-AROMA v0.3) (6). The resulting cleaned data set was detrended (up to a third-degree polynomial). Coregistration to a high-resolution T1 image (FSL FLIRT using the BBR algorithm), and normalization to 2mm isotropic MNI standard space (ANTs) was carried out. Lastly, preprocessed and normalized resting-state data sets were resliced to 3mm isotropic voxels. Datasets with a mean framewise displacement (FD) > 0.5mm in more than 10% of volumes were removed from further analysis, resulting removal of 5.1% of scans. Mean FD was included as covariate of no interest in functional analyses.

Psychopathology assessment

Psychopathology was assessed by self-report on the Strengths and Difficulties Questionnaire (SDQ), a 25-item scale assessing behavioural and emotional difficulties, administered at each study timepoint. The SDQ comprises five subscales: emotional symptoms, conduct problems, hyperactivity/inattention, peer relationship problems, and prosocial behaviour. Each item is rated on a 3-point scale. Following standard scoring procedures, total difficulties scores were derived by summing the four difficulties subscales. Internalising scores were calculated as the sum of emotional symptoms and peer problems subscales, and externalising scores as the sum of conduct problems and hyperactivity/inattention subscales. Total difficulties scores, internalising scores, and externalising scores were derived for all participants at each timepoint (age 14, 19, and 23).

Marginal modelling with FSL-SWE

Skeletonised diffusion metric maps (FA, TR, RD, AD) and functional connectivity maps (DC) underwent separate but identical voxelwise longitudinal analyses using nonparametric mixed-effects marginal modelling with the FSL Sandwich Estimator (FSL-SwE) tool (7), testing the longitudinal development of each brain metric and its association with psychopathology. FSL-SwE accommodates unbalanced data and includes all available observations. While participants with only one timepoint contribute to between-subject (cross-sectional) effects, only those with at least two timepoints contribute to within-subject (longitudinal) estimates. Similarly to random effects in linear mixed effects modelling, SwE treats participant as the clustering variable, thereby accounting for the non-independence of repeated measures within individuals via cluster-robust variance estimation. This approach adjusts the standard errors for within-participant correlation without requiring specification of a random-effects structure. This approach is computationally efficient and well suited for large, multi-site, unbalanced longitudinal neuroimaging datasets.

Sensitivity analyses

Additional sensitivity analyses were run to test the robustness of the observed effects. For each significant finding reported below, the mean of the relevant imaging metric (FA or DC) was extracted across significant clusters and submitted to linear mixed effects (LME) modelling in RStudio, including all terms equivalent to the whole-brain SwE analysis, with participant as random effect. Type III ANOVA was performed on LME models to replicate significant effects of interest from the SwE models, and to subsequently test whether these effects remained significant under alternative model specifications, see below.

Three sensitivity analyses were conducted: 1) Including a measure of socio-economic status (SES), as measured by the “family stress / socio-economic status” subscale of the Development and Well-Being Assessment (DAWBA) assessed at baseline (age 14), as additional covariate; 2) including additional sex-moderated effects of age, time and psychopathology-related terms given documented sex differences in psychopathology emergence and trajectories in adolescence, and 3) applying an additional, more stringent exclusion criterion to the functional imaging analysis (mean FD >0.2mm).

Replication of effects of interest in extracted ROIs

For the diffusion data, initial LME models including equivalent terms to whole-brain SwE models replicated a significant positive effect of Age on mean FA in the ROI covering much of the white matter skeleton ($\chi^2(1) = 423.67$, $p < .0001$), a negative effect of Age in the bilateral corticospinal tract (CST) ROI ($\chi^2(1) = 113.49$, $p < .0001$), a positive effect of Time in large parts of the white matter skeleton ($\chi^2(1) = 1555.40$, $p < .0001$), and a negative effect of Time in bilateral corticospinal tract (CST) ($\chi^2(1) = 398.33$, $p < .0001$). The negative effect of cross-sectional SDQ-TOT across much of the white matter skeleton was observed ($\chi^2(1) = 29.36$, $p < .0001$), as well as that of cross-sectional SDQ-EXT ($\chi^2(1) = 10.92$, $p < .001$). Finally, the negative time × cross-sectional SDQ-EXT interaction in left CST was significant ($\chi^2(1) = 24.09$, $p < .0001$).

For the resting-state data, LME results replicated the significant effect of longitudinal SDQ-TOT on DC in bilateral posterior superior frontal gyri (pSFG) ($\chi^2(1) = 27.63$, $p < .0001$), as well

as the significant cross-sectional age X cross-sectional SDQ-TOT interaction in left inferior temporal gyrus (ITG) ($\chi^2(1) = 24.68$, $p < .0001$).

Controlling for SES

All models were re-run while controlling for socio-economic status (SES) at baseline, as measured by the "family stress / socio-economic status" scale of the Development and Well-Being Assessment (DAWBA) at age 14.

After controlling for SES, all observed effects of interest remained significant.

Specifically, the positive ($\chi^2(1) = 413.74$, $p < .0001$) and negative ($\chi^2(1) = 13.00$, $p < .0001$) effects of Age, as well as the positive ($\chi^2(1) = 1546.2$, $p < .0001$) and negative ($\chi^2(1) = 399.14$, $p < .0001$) effects of Time on FA remained significant. The negative effect of cross-sectional SDQ-TOT ($\chi^2(1) = 25.47$, $p < .0001$) and cross-sectional SDQ-EXT ($\chi^2(1) = 8.23$, $p = .004$) remained significant. Finally, the negative time × cross-sectional SDQ-EXT interaction in left CST remained significant ($\chi^2(1) = 23.92$, $p < .0001$). Similarly for the functional analysis, the effect of longitudinal SDQ-TOT on DC in bilateral pSFG remained significant ($\chi^2(1) = 26.22$, $p < .0001$), as well as the cross-sectional age X cross-sectional SDQ-TOT interaction in left ITG ($\chi^2(1) = 24.96$, $p < .0001$).

Controlling for sex-moderated effects

All models were subsequently re-run after inclusion of additional sex-moderated effects. It is worth noting that these were not included as effects of interest, but rather to evaluate whether the primary findings were robust to potential moderation by sex. Specifically, the following interaction terms were added to each model: Age × sex, Time × Sex, cross-sectional SDQ (TOT, EXT, and/or INT) × sex, Age × cross-sectional SDQ × sex, and Time × cross-sectional SDQ × sex.

After inclusion of these interaction terms, all observed effects of interest remained significant.

Specifically, the positive ($\chi^2(1) = 387.04$, $p < .0001$) and negative ($\chi^2(1) = 123.17$, $p < .0001$) effects of Age, as well as the positive ($\chi^2(1) = 145.67$, $p < .0001$) and negative ($\chi^2(1) = 385.50$, $p < .0001$) effects of Time on FA remained significant. The negative effect of cross-sectional SDQ-TOT ($\chi^2(1) = 28.43$, $p < .0001$) and cross-sectional SDQ-EXT ($\chi^2(1) = 14.44$, $p < .001$) remained significant. Finally, the negative time × cross-sectional SDQ-EXT interaction in left CST remained significant ($\chi^2(1) = 23.39$, $p < .0001$). Similarly for the functional analysis, the effect of longitudinal SDQ-TOT on DC in bilateral pSFG remained significant ($\chi^2(1) = 27.74$, $p < .0001$), as well as the cross-sectional age X cross-sectional SDQ-TOT interaction in left ITG ($\chi^2(1) = 26.91$, $p < .0001$).

Additional motion exclusions

Finally, functional models were re-run after excluding additional participants based on a more stringent motion exclusion criterion of mean framewise displacement (FD) of > 0.2mm (in addition to the original exclusion criterion of FD>0.5mm in more than 10% of volumes), resulting in exclusion of an additional 71 scans from 23 participants. After excluding these participants, all observed effects of interest remained significant.

Specifically, the effect of longitudinal SDQ-TOT on DC in bilateral pSFG remained significant ($\chi^2(1) = 28.89$, $p < .0001$), as well as the cross-sectional age X cross-sectional SDQ-TOT interaction in left ITG ($\chi^2(1) = 25.31$, $p < .0001$).

Table S1. Developmental effects on fractional anisotropy. Only clusters larger than 100 voxels are reported.

Effect	Cluster index	Cluster size (voxels)	Peak MNI coordinates			*p* (TFCE-corrected)	Peak label (JHU ICBM-DTI-81 White Matter atlas)
**Positive effect of cross-sectional age**	1	22785	41	9	-34	<.001	Inferior longitudinal fasciculus (L)
	2	3709	-47	-6	-31	<.001	Inferior longitudinal fasciculus (R)
	3	2678	-43	39	-10	<.001	Inferior fronto-occipital fasciculus (R)
	4	2624	-16	58	-9	<.001	Inferior fronto-occipital fasciculus (R)
	5	2307	-38	-39	21	<.001	Superior longitudinal fasciculus (R)
	6	536	-21	19	1	<.001	Anterior limb of the internal capsule (R)
	7	519	-6	-23	-14	<.001	Unclassified
	8	428	-26	-77	27	<.001	Inferior fronto-occipital fasciculus (R)
	9	363	32	18	39	<.001	Unclassified
	10	320	-14	-47	-36	<.001	Unclassified
	11	270	-27	39	-4	<.001	Inferior fronto-occipital fasciculus (R)
	12	195	-10	-36	31	<.001	Cingulum (cingulate) (R)
	13	190	-14	-56	27	<.001	Unclassified
	14	178	-38	18	44	<.001	Unclassified
	15	165	9	-37	32	<.001	Cingulum (cingulate) (L)
	16	137	6	-55	-32	<.001	Unclassified
	17	136	-37	32	24	.002	Unclassified
	18	118	-22	-36	29	.009	Anterior thalamic radiation (R)
	19	104	16	-39	-6	.005	Cingulum (hippocampal) (L)
**Negative effect of cross-sectional age**	1	398	-22	-16	-3	<.001	Posterior limb of the internal capsule (R)
	2	136	-27	-18	22	.004	Superior corona radiata (R)
**Positive effect of longitudinal age**	1	59538	-13	-42	-44	<.001	Unclassified
	2	608	24	-25	-26	<.001	Unclassified
	3	410	-28	-29	-6	<.001	Fornix (R)
	4	401	-18	-65	-42	<.001	Unclassified
	5	398	-27	-24	-26	<.001	Unclassified
	6	345	-11	-9	6	<.001	Unclassified
	7	207	-11	-35	31	<.001	Unclassified
	8	201	7	17	27	<.001	Cingulate (cingulate) (L)
	9	195	13	-31	-43	<.001	Unclassified
	10	139	-27	-61	-2	<.001	Unclassified
**Negative effect of longitudinal age**	1	2131	-18	-19	-13	<.001	Cerebral peduncle (R)
	2	603	23	-32	34	<.001	Posterior corona radiata (L)
	3	214	-34	5	-37	<.001	Unclassified
	4	121	21	-15	4	<.001	Posterior limb of the internal capsule (L)

Table S2. Negative effect of cross-sectional SDQ-TOT on fractional anisotropy. Only clusters larger than 100 voxels are reported.

Cluster index	Cluster size (voxels)	Peak MNI coordinates			*p* (TFCE-corrected)	Peak label (JHU ICBM-DTI-81 White Matter atlas)
1	5252	3	-38	-38	<.001	Medial lemniscus (L)
2	3489	-12	-40	10	<.001	Corpus callosum (splenium)
3	1518	-36	-39	10	.002	Inferior fronto-occipital fasciculus (R)
4	1442	10	-84	-63	<.001	Inferior longitudinal fasciculus (L)
5	1095	10	31	-1	.002	Corpus callosum (genu)
6	722	-16	36	-1	.005	Anterior corona radiata (R)
7	233	14	-66	42	.009	Unclassified
8	160	25	-75	13	.004	Unclassified

Table S3. Negative effect of cross-sectional SDQ-EXT on fractional anisotropy. Only clusters larger than 100 voxels are reported.

Cluster index	Cluster size (voxels)	Peak MNI coordinates			*p* (TFCE-corrected)	Peak label (JHU ICBM-DTI-81 White Matter atlas)
1	3146	-7	-38	-39	<.001	Medial lemniscus (R)
2	3079	37	-47	2	<.001	Posterior thalamic radiation (L)
3	2261	-13	-40	9	<.001	Corpus callosum (splenium)
4	1828	-37	-39	7	<.001	Posterior thalamic radiation (R)
5	440	11	32	-2	<.001	Corpus callosum (genu)
6	340	-14	34	-2	.002	Corpus callosum (genu)
7	252	-45	-7	-26	.017	Unclassified
8	242	-43	-42	-6	.010	Unclassified
9	215	18	-34	-12	<.001	Cingulum (hippocampal) (L)

Table S4. Negative effect of time × cross-sectional SDQ-EXT on fractional anisotropy.

Cluster index	Cluster size (voxels)	Peak MNI coordinates			*p* (TFCE-corrected)	Peak label (JHU ICBM-DTI-81 White Matter atlas)
1	79	23	-20	7	.005	Posterior limb of the internal capsule (L)

Table S5. Main effect of longitudinal SDQ-TOT on degree centrality.

Cluster index	Cluster size (voxels)	Peak MNI coordinates			*p* (TFCE-corrected)	Peak label (Harvard-Oxford Atlas)
1	33	24	-12	51	.038	Superior frontal gyrus (R)
2	32	-18	-6	63	.041	Superior frontal gyrus (L)

Table S6. Effect of cross-sectional age X cross-sectional SDQ-TOT on degree centrality.

Cluster index	Cluster size (voxels)	Peak MNI coordinates			*p* (TFCE-corrected)	Peak label (Harvard-Oxford Atlas)
1	26	-51	-36	-24	.043	Inferior frontal gyrus (L)

Figure S1. Histogram of mean subject age across timepoints.

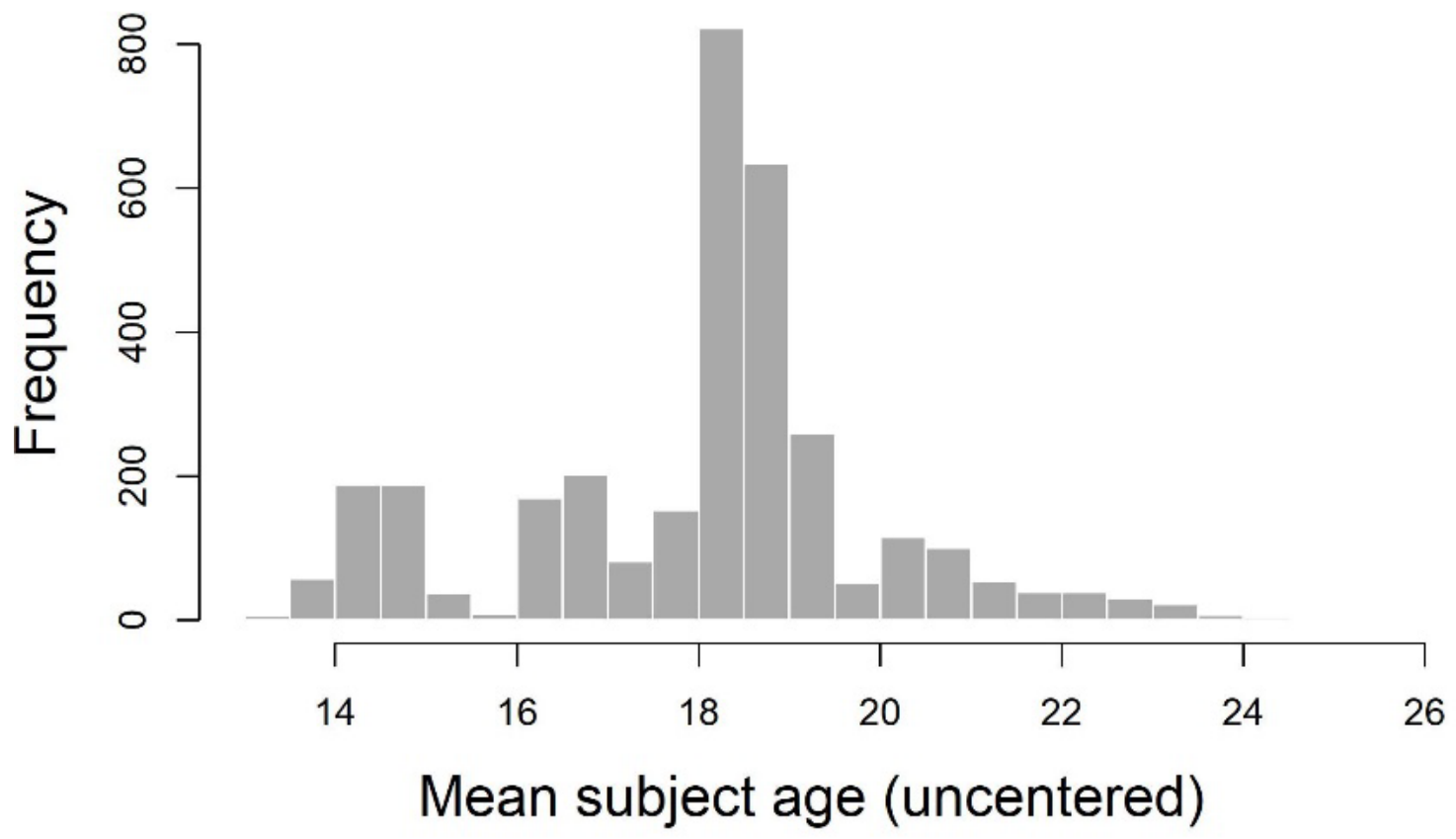

Figure S2. Positive (red) and negative (blue) developmental effects on trace (TR), radial diffusivity (RD), and axial diffusivity (AD), captured cross-sectionally (A) and longitudinally (B).

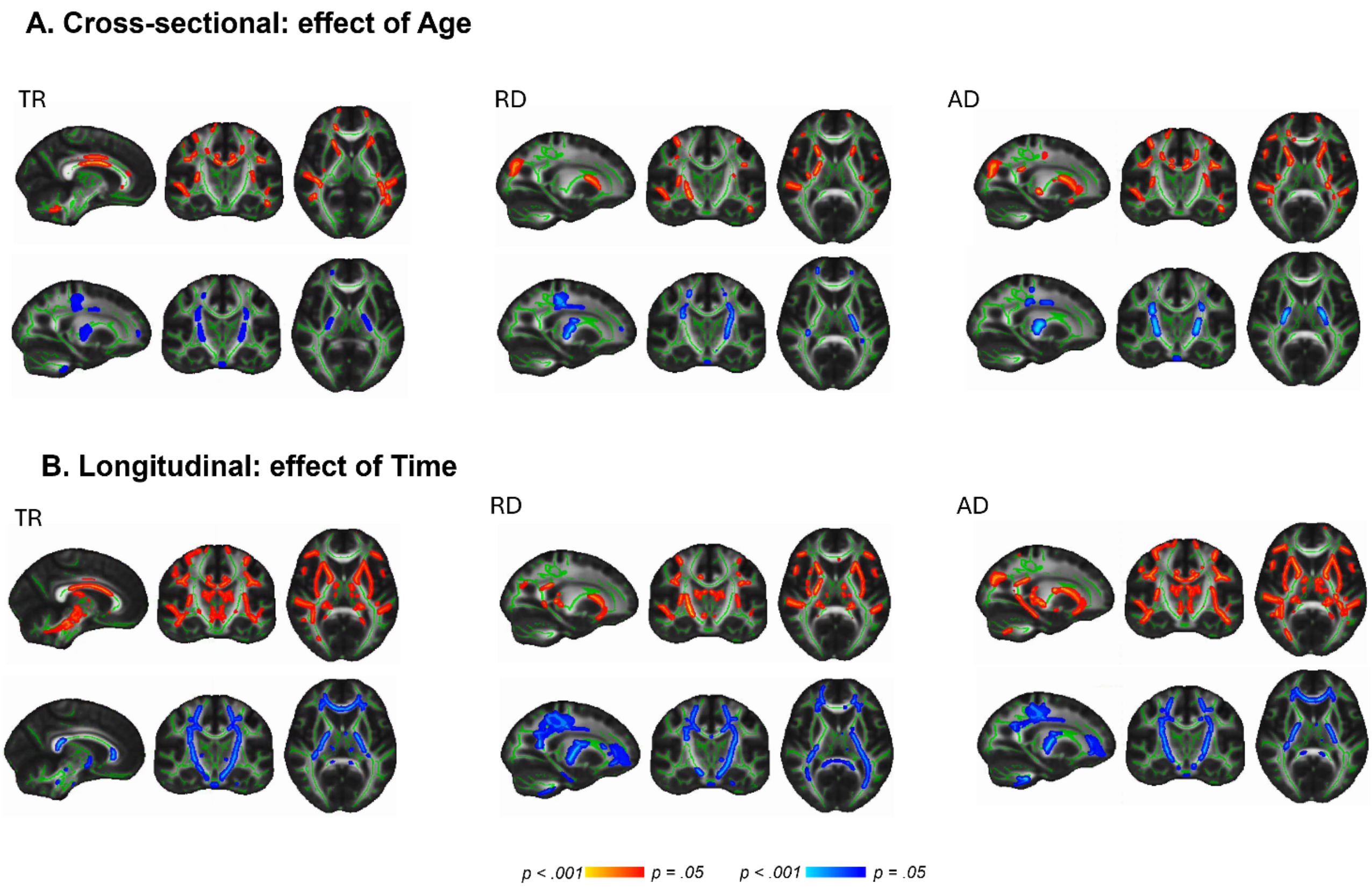

Table S7. Effect of site on imaging metrics

	ANOVA		
**Measure**	***F***	***df***	***p***
Mean FA (age 14)	166.4	(7, 1403)	<.001
Mean FA (age 19)	142.5	(7, 935)	<.001
Mean FA (age 23)	118.9	(7, 916)	<.001
Mean DC (age 14)	1.44	(4, 326)	.220
Mean DC (age 19)	1.67	(7, 980)	.113
Mean DC (age 23)	1.76	(7, 1107)	.091

Note: Mean fractional anisotropy (FA) is measured across the white matter skeleton. Mean degree centrality (DC) is measured across the grey matter.

References

1. Schumann G, Loth E, Banaschewski T, Barbot A, Barker G, Büchel C, et al. (2010): The IMAGEN study: reinforcement-related behaviour in normal brain function and psychopathology. *Molecular psychiatry*. 15:1128-1139.
2. Zhang H, Yushkevich PA, Alexander DC, Gee JC (2006): Deformable registration of diffusion tensor MR images with explicit orientation optimization. *Medical image analysis*. 10:764-785.
3. Keihaninejad S, Zhang H, Ryan NS, Malone IB, Modat M, Cardoso MJ, et al. (2013): An unbiased longitudinal analysis framework for tracking white matter changes using diffusion tensor imaging with application to Alzheimer's disease. *Neuroimage*. 72:153-163.
4. Zhang S, Arfanakis K (2018): Evaluation of standardized and study-specific diffusion tensor imaging templates of the adult human brain: Template characteristics, spatial normalization accuracy, and detection of small inter-group FA differences. *Neuroimage*. 172:40-50.
5. Smith SM, Jenkinson M, Johansen-Berg H, Rueckert D, Nichols TE, Mackay CE, et al. (2006): Tract-based spatial statistics: voxelwise analysis of multi-subject diffusion data. *Neuroimage*. 31:1487-1505.
6. Pruim RH, Mennes M, van Rooij D, Llera A, Buitelaar JK, Beckmann CF (2015): ICA-AROMA: A robust ICA-based strategy for removing motion artifacts from fMRI data. *Neuroimage*. 112:267-277.
7. Guillaume B, Hua X, Thomson P, Waldorp L, Nichols T (2014): The SwE Toolbox: a Toolbox for the Analysis of Longitudinal and Repeated Measures Neuroimaging Data.
